# The evolution of insecticide resistance in the brown planthopper (*Nilaparvata lugens* Stål) of China in the period 2012–2016

**DOI:** 10.1038/s41598-018-22906-5

**Published:** 2018-03-15

**Authors:** Shun-Fan Wu, Bin Zeng, Chen Zheng, Xi-Chao Mu, Yong Zhang, Jun Hu, Shuai Zhang, Cong-Fen Gao, Jin-Liang Shen

**Affiliations:** 1 College of Plant Protection, State & Local Joint Engineering Research Center of Green Pesticide Invention and Application, Nanjing Agricultural University, Nanjing, 210095 China; 2 National Agro-tech Extension and Service Center, Ministry of Agriculture, Beijing, 100125 China

## Abstract

The brown planthopper, *Nilaparvata lugens*, is an economically important pest on rice in Asia. Chemical control is still the most efficient primary way for rice planthopper control. However, due to the intensive use of insecticides to control this pest over many years, resistance to most of the classes of chemical insecticides has been reported. In this article, we report on the status of eight insecticides resistance in *Nilaparvata lugens* (Stål) collected from China over the period 2012–2016. All of the field populations collected in 2016 had developed extremely high resistance to imidacloprid, thiamethoxam, and buprofezin. Synergism tests showed that piperonyl butoxide (PBO) produced a high synergism of imidacloprid, thiamethoxam, and buprofezin effects in the three field populations, YA2016, HX2016, and YC2016. Functional studies using both double-strand RNA (dsRNA)-mediated knockdown in the expression of *CYP6ER1* and transgenic expression of *CYP6ER1* in *Drosophila melanogaster* showed that *CYP6ER1* confers imidacloprid, thiamethoxam and buprofezin resistance. These results will be beneficial for effective insecticide resistance management strategies to prevent or delay the development of insecticide resistance in brown planthopper populations.

## Introduction

The brown planthopper (BPH), *Nilaparvata lugens* (Stål) (Hemiptera: Delphacidae), is a serious pest on rice in Asia^1^. This monophagous pest causes severe damage to rice plants through direct sucking often causing “hopper burn”, ovipositing and virus disease transmission during its long-distance migration^1,2^. In recent years, *N*. *lugens* outbreaks have occurred more frequently in China and other Asian countries^3^. The damage to the rice crop can result in a significant loss of yield in susceptible rice varieties^1^. The losses to rice production caused by *N*. *lugens* in Asia have been estimated as more than $300 million annually^4^.

The control of BPH has for many years predominantly relied on the use of synthetic chemicals^5^. However, due to the large scale and intensive use of insecticides, BPH has evolved high levels of resistance to many of the major classes of insecticides including organophosphates, carbamates, pyrethroids, neonicotinoids, insect growth regulators, and phenylpyrazoles^3,6^. Since the early 1990s, the neonicotinoid insecticide imidacloprid was introduced into Asia for BPH control^7^. Resistance to this insecticide emerged in field populations across Asia over the period 2005–2012^7,8^. Following suspension of imidacloprid in 2006, banning of fipronil and buprofezin in 2009 and 2013, respectively, for controlling *N*. *lugens* in China, pymetrozine, thiamethoxam, flufiprole, nitenpyram, dinotefuran, sulfoxaflor and chlorpyrifos have been commonly used for controlling this pest insect in China in recent years^9^. BPH has developed resistance to 29 compounds in the world^10^. Therefore, resistance monitoring is a key to understand the current status of susceptibility of the field population of *N*. *lugens* to various insecticides^6^. Early detection of changes in resistance/susceptibility can prompt adoption of alternative control measures, which are essential for the successful management of this pest^3^.

The mechanisms of insect resistance to insecticides involve the over-expression or mutations of detoxifying enzyme genes and amino acid mutations of targeted genes^10,11^. The molecular mechanism(s) underlying resistance to imidacloprid have been characterised in the BPH. Although target-site resistance to this compound was described in a laboratory-selected strain of BPH, this mutation site has never been detected in filed-collected population^5,12–14^. In contrast, it was generally accepted that, changes in detoxifying enzymes, especially enhanced cytochrome P450 monooxygenase (P450) activity contributes to the neonicotinoid resistance of field-collected populations of BPH^5,15–19^. This detoxification mechanism was initially implicated by use of the metabolic enzyme inhibitor piperonyl butoxide (PBO) and the model substrate 7-ethoxycoumarin^13,14^. Although many P450 genes were over-expressed in resistant strains or field populations of BPH, two candidate P450 gens, *CYP6ER1* and *CYP6AY1*, has been linked with imidacloprid resistance^5,16,18,20^. It was reported that *CYP6AY1* was identified as the highest level of overexpression in the resistant strain compared with the susceptible strain and functional expression of *CYP6AY1* proved that *CYP6AY1* has the capacity to metabolise imidacloprid^15^. While one study showed that *CYP6AY1* was not over-expressed even down-regulated in most of imidacloprid resistance field populations^5^. Recent studies showed that *CYP6AY1* metabolized imidacloprid more efficiently and *CYP6ER1* gene could be up-regulated by imidacloprid at a higher level and metabolic imidacloprid resistance in BPH relies on multiple P450 enzymes^16,21^. It was reported that the cross-resistance between imidacloprid and thiamethoxam was presented in the imidacloprid-resistant strains of *N*. *lugens*^22,23^. However, little is known about the resistance mechanism of BPH to thiamethoxam and buprofezin.

The aim of this study was to provide the changing levels of resistance to eight insecticides in *N*. *lugens* field strains collected from eight provinces in China from 2012 to 2016, and to study the relative roles of the cytochrome P450 monooxygenase (P450), *CYP6ER1* and *CYP6AY1*, in determining their resistance phenotype to imidacloprid, thiamethoxam and buprofezin.

## Results and Discussion

### Variations in resistance ratios to eight insecticides

**Figure 1:**
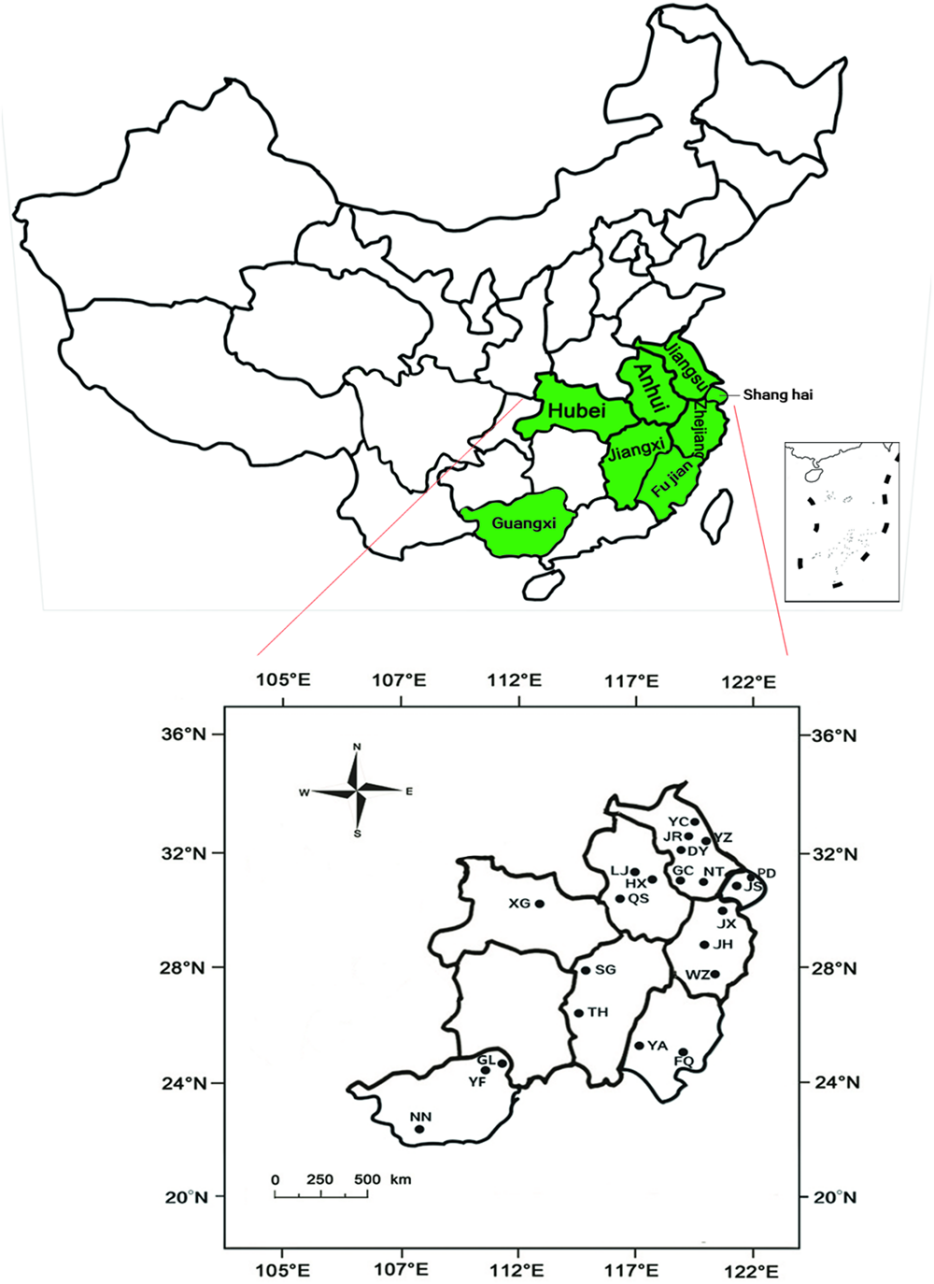
Collection sites of *Nilaparvata lugens* populations during 2012–2016. The map in this figure were generated by software Adobe Photoshop CS5 version (San Jose, CA, http://www.adobe.com/products/photoshop.html) based on our own data.

**Figure 2:**
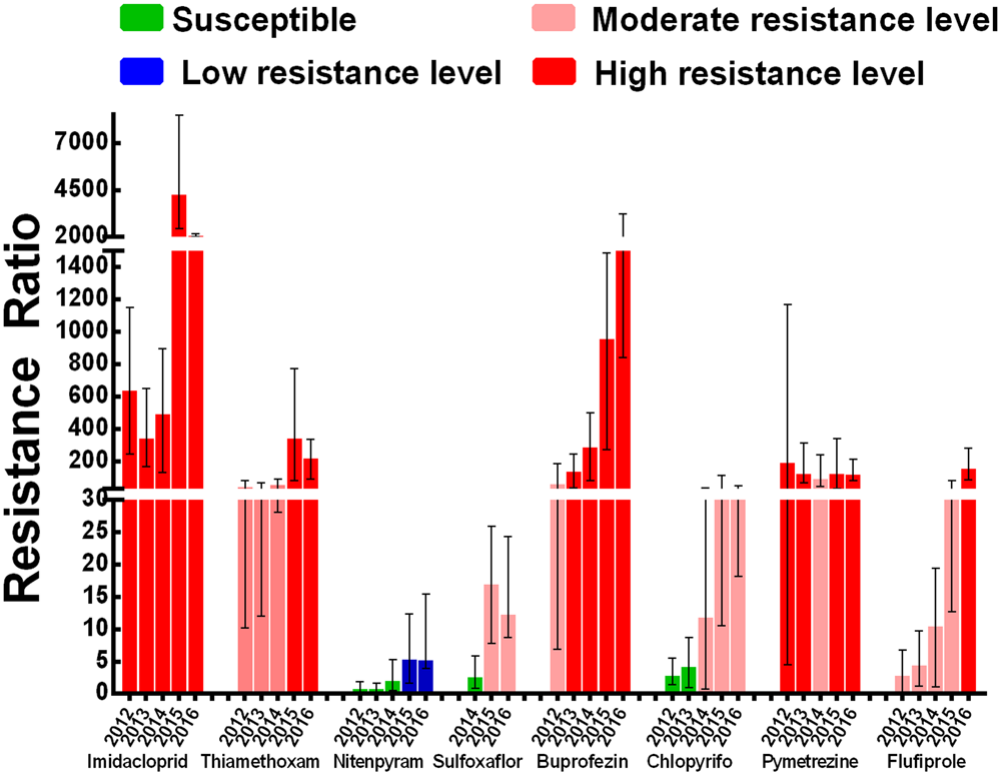
Resistance ratios to eight insecticides of the mixed population in each year. The data represents means and upper or lower limit.

Resistance to eight insecticides were monitored in sixty-nine field populations of *N*. *lugens* collected from eight Chinese provinces in the period 2012–2016 (Supplementary Table S1 and Fig. 1). Pooled resistance ratio (RR) data from all populations of the same year indicated that substantially different resistance levels were developed in *N*. *lugens* to eight different insecticides (Fig. 2). The increase in RR over years was observed in all of insecticides. Our results indicated that *N*. *lugens* have developed high resistance levels to imidacloprid (mean RR value in 2016, 2104-fold), buprofezin (mean RR value in 2016, 1736-fold), thiamethoxam (mean RR value in 2016, 222-fold), pymetrozine (mean RR value in 2016, 125-fold) and flufiprole (mean RR value in 2016, 160-fold), a medium resistance level to chlorpyrifos (mean RR value in 2016, 30-fold) and sulfoxaflor (mean RR value in 2016, 12-fold) and a low resistance levels to nitenpyram (mean RR value in 2016, 5-fold). The results showed that resistance levels of six insecticides imidacloprid, thiamethoxam, nitenpyram, sulfoxaflor, buprofezin and flufiprole have a dramatic increase in 2015 and 2016 compared with former three years (Supplementary Table S4 and Fig. 2) and previously reported^6,24^.

### Nicotinc acetylcholine receptor (nAChR) competitive modulators: imidacloprid, thiamethoxam, nitenpyram and sulfoxaflor

**Figure 3:**
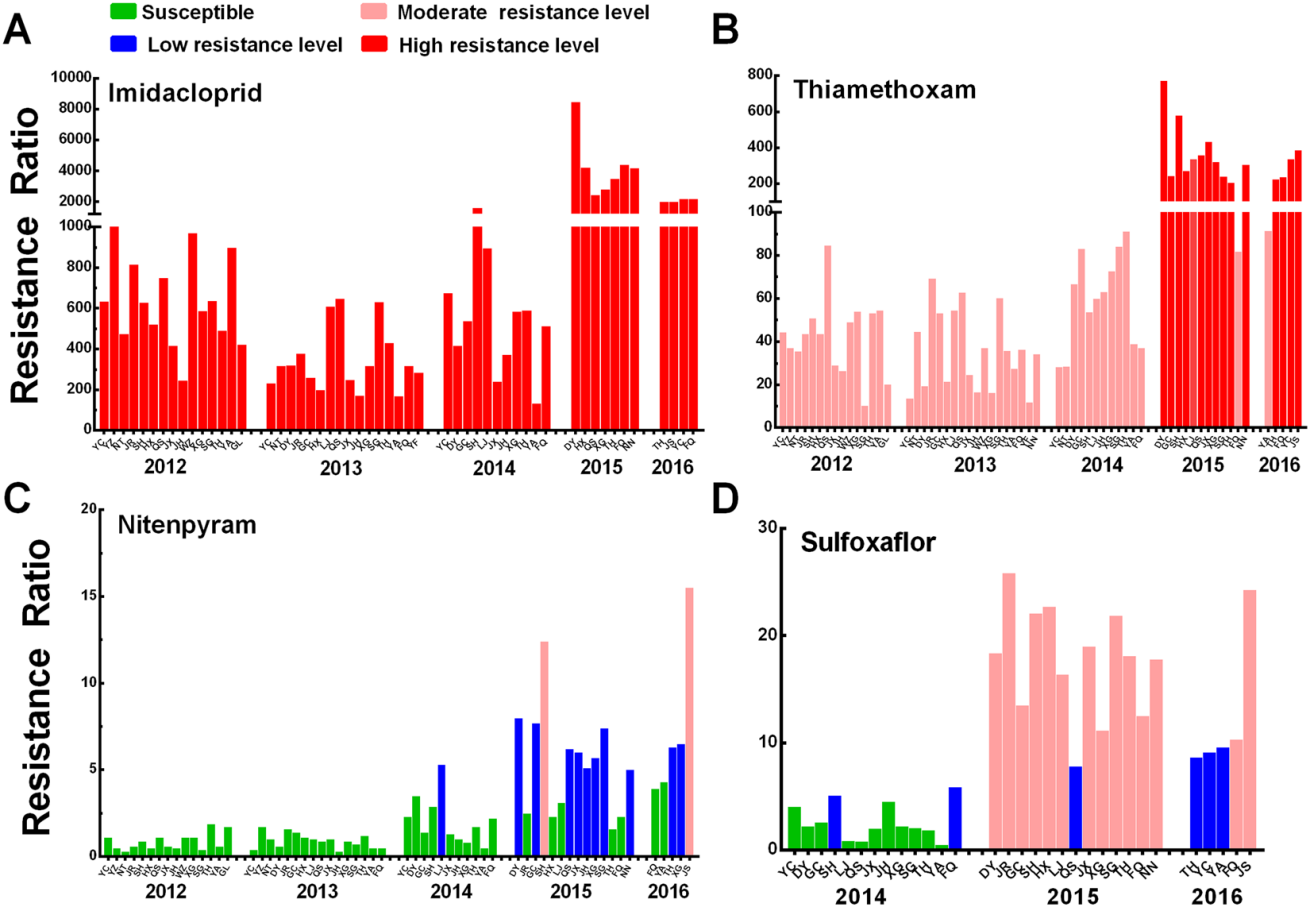
Resistance ratios to four nicotinic acetylcholine receptor (nAChR) competitive modulators, imidacloprid (**A**), thiamethoxam (**B**), nitenpyram (**C**) and sulfoxaflor (**D**) of *Nilaparvata lugens* populations during 2012–2016.

High to extremely high resistance levels to imidacloprid (RR: 132-8478-fold) and medium to high resistance levels to thiamethoxam (RR: 10-774-fold) were found in the field populations of *N*. *lugens* in 2012 to 2016 (Fig. 3A and B). The resistance ratio of *N*. *lugens* to imidacloprid and thiamethoxam showed an sharply increased in 2015. Surveys conducted in 1996–2007 showed that most populations of filed collected brown planthopper in China quickly developed medium to high level of resistance to imidacloprid in 2005–2006^7,19^. Similar phenomenon was also observed by other studies^5^. Hence, suspension of imidacloprid for controlling *N*. *lugens* was carried out in China since 2006^7^. However, the resistance of *N*. *lugens* to imidacloprid did not decrease in recent years. One reason is that imidacloprid is being used for controlling white-backed planthopper in China^25^. This will make BPH contact with imidacloprid. Another possible reason is, in other Southeast Asian countries, the intensive use of imidacloprid against *N*. *lugens*, which subsequently migrate to China^22^. Since *N*. *lugens* has evolved high level of resistance to imidacloprid, thiamethoxam has been used for BPH control instead of imidacloprid. Besides its extensive and intensive use, the main reason for the rapid resistance of *N*. *lugens* development to thiamethoxam might be the cross-resistance between imidacloprid and thiamethoxam in the imidacloprid-resistant strains of *N*. *lugens*^22,23^.

Most field populations developed a low to medium resistance level to nitenpyram (5–12.4-fold), except five populations, which were still susceptible or slightly resistant to the chemical in 2015 (Fig. 3C). However, one population (JS2016) have developed medium resistance levels (15.5-fold) to nitenpyram. One recent study also showed that nitenpyram resistance has appeared in some field populations of *N*. *lugens* in China and cytochrome P450 monooxygenase is likely a contributing factor to this insecticide resistance^26^. Sulfoxaflor, a new developed chemical by Dow AgroSciences, exhibits broad-spectrum control of many sap-feeding pests, including planthoppers, aphids, whiteflies and true bugs^27,28^. The susceptibility of *N*. *lugens* to sulfoxaflor was only investigated in three years. However, to our surprise, the results showed that the populations of *N*. *lugens* collected in 2015 and 2016 have developed low to medium resistance levels to sulfoxaflor (7.8–25.9-fold) (Fig. 3D). Liao *et al*. also showed that field populations of *N*. *lugens* collected in 2016 have developed a low level of resistance to sulfoxaflor^29^. These results imply that these two neonicotinoid insecticides, nitenpyram and sulfoxaflor, might have a potentially risk of resistance to *N*. *lugens*^30^.

### Inhibitors of chitin biosynthesis: buprofezin

**Figure 4:**
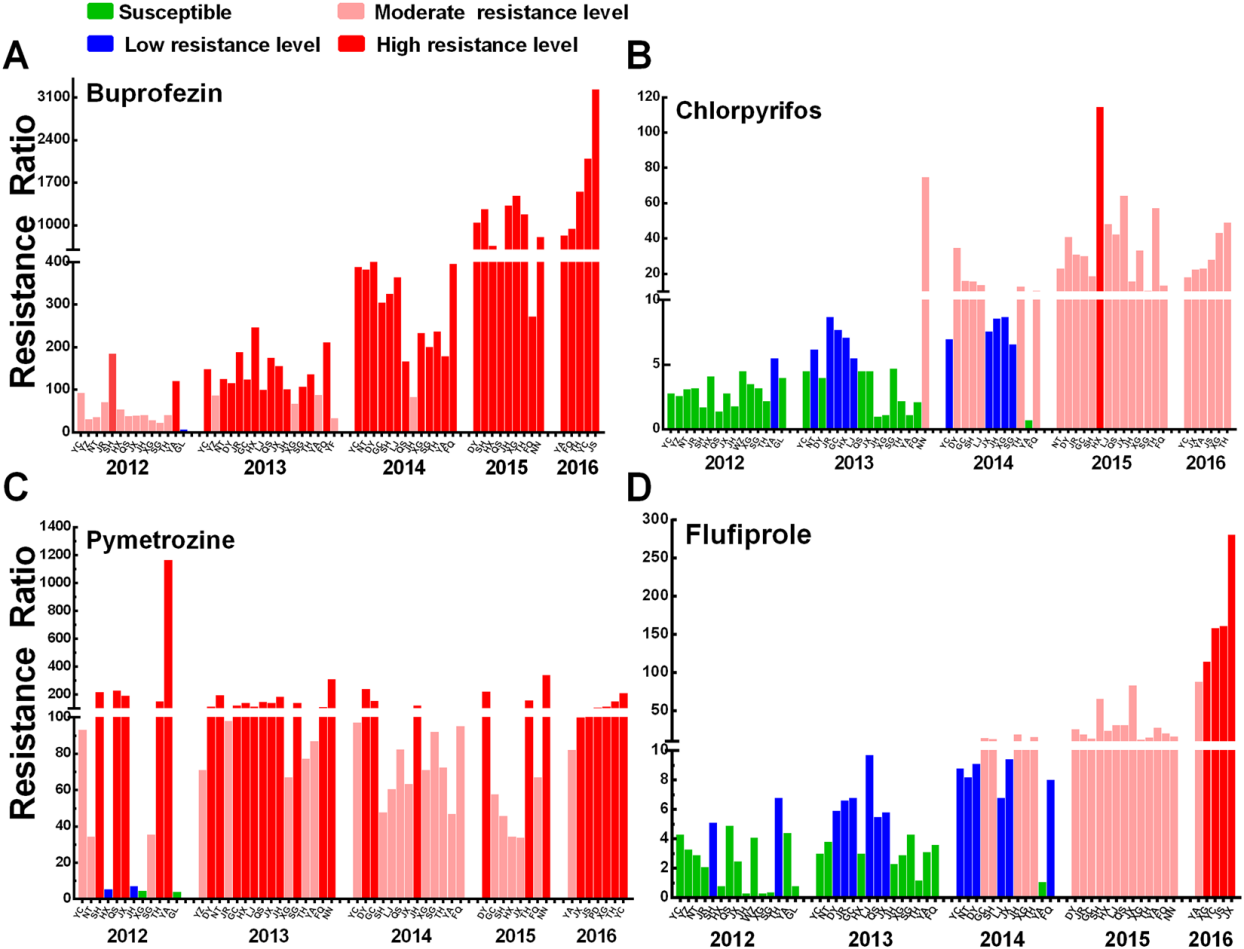
Resistance ratios to buprofezin (**A**), chlorpyrifos (**B**), pymetrozine (**C**) and flufiprole (**D**) of *Nilaparvata lugens* populations during 2012–2016.

Of the 14 field populations of BPH collected in 2012 in China, 11 field populations were at medium resistance level to buprofezin (24–93-fold) except SH2012 (Shanghai) and YA2012 (Yongan) populations, which had reached to high resistance level (102 and 154-fold). And there was one population (GL2012) maintained susceptibility (RR < 5-fold) to the insecticide. However, in 2016, although the resistance ratios were substantially different among the populations, all populations had reached to high resistance level (839–3241.5-fold) to buprofezin (Fig. 4A). Buprofezin has been used for many years to control *N*. *lugens* in China. Most field populations of *N*. *lugens* were susceptible before 2004. However, resistance to buprofezin had been found in some field populations of *N*. *lugens* after 2004^19^. And medium to high level of resistance was observed in 2010–2012 in China^9,24^. Our monitoring results suggested that the field populations of *N*. *lugens* have developed high levels of resistance to buprofezin. For this reason, it was suspended for the control of *N*. *lugens* in 2014 by the National Agro-tech Extension and Service Center, Ministry of Agriculture of China based on our results. However, the main reason why the resistance to buprofezin sharply increased in 2015 and 2016 need addressed in the future.

### Acetylcholinesterase (AChE) Inhibitors: chlorpyrifos

The results showed that populations collected in 2012 kept susceptibility to chlorpyrifos except YA2012 population, which developed low resistance levels to chlorpyrifos. The surveys conducted in the next three years showed that the resistance ratio relatively increased every year. In 2015, all of the populations developed medium resistance levels to chlorpyrifos (RR 13.5–64.3-fold) except one population (HX2015), which developed high resistance level to chlorpyrifos (110-fold) (Fig. 4B). Because *N*. *lugens* had developed high level of resistance to insecticides such as imidacloprid, buprofezin, and thiamethoxam in the field in recent years, some farmers chose organophosphorus and carbamates insecticides for controlling brown planthopper^6,31^. Therefore, the resistance in this insect to chlorpyrifos increased every year in China^6^.

### Chordotonal organ Transient Receptor Potential Vanilloid (TRPV) channel modulators: pymetrozine

The resistance ratios of *N*. *lugens* to pymetrozine in 2012 had a greater difference (4–1168-fold). In 2013, 68.8% of populations had a high resistance level (118–313-fold) and the others maintained a medium resistance level (67–98-fold), consistent with previously reported^9,24^. In 2014 and 2015, pymetrozine resistance of most populations decreased compared to the level in the previous two years. In 2015, there are two populations showed low resistance level (QS2015, 5-fold) or susceptible to pymetrozine (XG2015, 3.5-fold). Three populations were at high levels to pymetrozine (160, 223 and 340-fold) (Fig. 4C). However, in 2016, most of populations except YA2016, have developed high level of resistance to pymetrozine (Fig. 4C). Pymetrozine disrupt coordination and feeding of plant-sucking insects and are effective against insects that have developed to other insecticides^32^. Its molecular target have been identified as a transient receptor potential (TRP) ion channel complex^33^. The pymetrozine resistance was associated with increasing use of this insecticide against brown planthopper in China and Southeast Asian countries. As reported in the greenhouse whitefly, another possible reason was cross-resistance between pymetrozine and neonicotinoid insecticides^34^.

### GABA-gated chloride channel blockers: flufiprole

Flufiprole is a novel kind of phenylpyrazole insecticide developed by Dalian Raiser Pesticide Co., Ltd., China^35^. Most populations of *N*. *lugens* collected in 2012 were susceptible to flufiprole except SH2015 (Shanghai) and TH2015 (Taihe) populations, which developed low level of resistance. However, there were clear increase of resistance in the next four years compared to results of 2012. In 2016, all of the populations have evolved medium to high level of resistance to flufiprole (88–281-fold) (Fig. 4D). Flufiprole have not been used extensively to control *N*. *lugens* in the rice-growing areas in China. In 2012–2013, field populations of *N*. *lugens* remain susceptible to flufiprole. However, it was recommended for *N*. *lugens* control instead of those insecticides to which the pest has developed high resistance levels, such as imidacloprid or buprofezin. Hence, in 2014–2015, *N*. *lugens* has developed low to moderate levels of resistance to flufiprole. The other possible reason was the cross-resistance between flufiprole and ethiprole or fipronil, which were used intensively and extensively against *N*. *lugens* in immigrant source areas such as Thailand^5,6,36^.

### Synergists assessment

The synergistic effects of three representative synergists, piperonyl butoxide (PBO), triphenyl phosphate (TPP) and diethyl meteate (DEM), on imidacloprid, thiamethoxam, and buprofezin toxicities in three field populations were tested (Table 1). In the susceptible strain (SS), none of three synergists showed significant synergism on imidacloprid, thiamethoxam, and buprofezin. However, PBO significantly synergized imidacloprid, thiamethoxam, and buprofezin in field populations with the synergism ratio (SR) of 4.1-, 2.8-, and 3.5-fold, respectively, suggesting cytochrome P450s are involved in the resistance of BPH to imidacloprid, thiamethoxam, and buprofezin (Table 1). Cytochrome P450s have been reported that are the primary enzyme system involved in the resistance of brown planthopper to imidacloprid^5,16,21,37^. Previous studies have proved that insecticides cross-resistance was presented between imidacloprid, thiamethoxam, and buprofezin in field populations of BPH^6,23^, peach-potato aphid^38^ and Colorado potato beetle^39^. Hence, the results suggested that cytochrome P450 monooxygenases might play an important role in the resistance of *N*. *lugens* to imidacloprid, thiamethoxam, and buprofezin.

**Table 1:** Synergistic effects of DEM, TPP and PBO on susceptible strain, YA2016, HX2016 and YC2016 field populations to imidacloprid, thiamethoxam and buprofezin.

Populations	Treatment	Slope ± SE	LC_50_ (95%F.L.) (mg/L)	RR	SR^a^
SS	Imidacloprid	1.82 ± 0.33	0.28 (0.23–0.35)	1.0	
	+DEM	2.24 ± 0.38	0.32 (0.27–0.41)	1.1	0.9
	+TPP	2.58 ± 0.40	0.27 (0.22–0.34)	1.0	1.0
	+PBO	1.77 ± 0.32	0.19 (0.16–0.22)	0.7	1.5
SS	Thiamethoxam	1.81 ± 0.24	0.20 (0.16–0.26)		
	+DEM	1.94 ± 0.35	0.19 (0.15–0.24)	1.0	1.1
	+TPP	1.48 ± 0.35	0.18 (0.12–0.24)	0.9	1.1
	+PBO	1.28 ± 0.31	0.14 (0.07–0.21)	0.7	1.4
SS	Buprofezin	1.89 ± 0.26	0.97 (0.75–1.23)	1.0	
	+DEM	2.17 ± 0.29	0.73 (0.57–0.91)	0.8	1.3
	+TPP	1.93 ± 0.27	0.65 (0.49–0.83)	0.7	1.5
	+PBO	1.97 ± 0.28	0.59 (0.44–0.75)	0.6	1.6
YA2016	Imidacloprid	1.63 ± 0.34	137.43 (102.05–211.10)	490.8	
	+DEM	1.32 ± 0.30	94.74 (65.90–141.82)	338.4	1.5
	+TPP	1.23 ± 0.31	75.22 (46.77–111.68)	268.6	1.8
	+PBO	1.68 ± 0.33	33.50 (23.59–44.40)	119.7	4.1
HX2016	Thiamethoxam	2.27 ± 0.32	24.76 (13.61–39.77)	122.0	
	+DEM	2.27 ± 0.38	17.04 (12.48–21.43)	83.9	1.5
	+TPP	2.05 ± 0.37	13.85 (10.45–17.89)	68.2	1.8
	+PBO	1.80 ± 0.34	8.71 (5.47–11.61)	42.9	2.8
YC2016	Buprofezin	1.95 ± 0.27	154.12 (120.52–195.37)	158.9	
	+DEM	1.76 ± 0.29	120.14 (91.64–163.48)	123.8	1.3
	+TPP	1.68 ± 0.23	86.34 (67.30–112.53)	89.0	1.8
	+PBO	1.65 ± 0.25	44.16 (30.969–58.075)	45.5	3.5

^a^Synergism ratio = LC_50_ of insecticides/LC_50_ of insecticides + synergist.

### Association of overexpression of CYP6ER1 with resistance to imidacloprid, thiamethoxam and buprofezin

**Figure 5:**
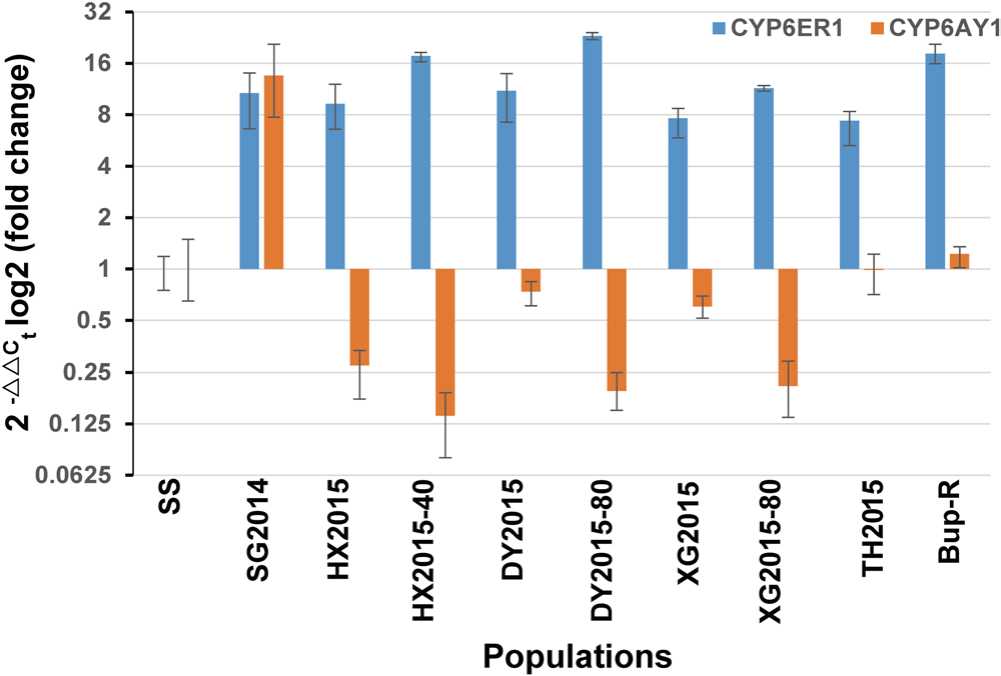
Fold change in expression of *CYP6ER1* and *CYP6AY1* in eight resistant *N*. *lugens* strains compared with the susceptible reference SS as determined by qPCR. Error bars display 95% fiducial limits.

Two cytochrome P450s (*CYP6ER1* and *CYP6AY1*) have previously been linked with imidacloprid resistance in a number of brown planthopper laboratory and field populations^5,15–17^. However, little is known about these two genes contributed to other insecticides resistance. In this present study, the mRNA levels of *CYP6ER1* and *CYP6AY1* were examined and compared in susceptible strain and five field populations collected from five areas in China that exhibited clear resistance to imidacloprid, thiamethoxam and buprofezin in discriminating dose bioassays and one buprofezin-resistance population (Bup-R, 533-fold). The results showed that *CYP6ER1* in all field populations and Bup-R strain were significantly overexpressed, when compared with a lab susceptible strain, with fold changes ranging from seven- to 24-fold. However, *CYP6AY1* was underexpressed in five of the populations compared with the same susceptible strain, and was only significantly overexpressed (13.5-fold) in a single population (SG2014) (Fig. 5).

To see whether selection of the field strains with thiamethoxam caused any increase in the expression levels of *CYP6ER1* or *CYP6AY1*, three field strains (HX2015, DY2015 and XG2015) were selected with thiamethoxam up to final concentrations of 40, 80 and 80 mg L^−1^ thiamethoxam, respectively. When the expression levels of *CYP6ER1* were compared between DY2015 (unselected) and DY2015-80 (selected), the expression level was found to have significantly increased two-fold after selection, rising from ∼11-to 23.2-fold. Similar effects were seen for HX2015 (unselected) versus HX2015-40 (selected) and XG2015 (unselected) versus XG2015-80 (selected). Besides this, the variation in the level of expression of *CYP6ER1* among individual biological replicates decreased considerably after selection (as showed by significantly reduced 95% fiducial limits – see Fig. 5). This indicated that thiamethoxam selection has reduced genetic heterogeneity in these strains and that all replicates overexpress *CYP6ER1* at a universally high level^5^. After selection, to our surprise, *CYP6AY1* expression significantly decreased from 0.27 in HX2015 to 0.14 in HX2015-40, from 0.74 in DY2015 to 0.20 in DY2015-80, and from 0.60 in XG2015 to 0.20 in XG2015-80 (Fig. 5).

Overexpression of *CYP6ER1* is associated with resistance to imidacloprid in the BPH^5,18,21^. Our results provide evidence that overexpression of *CYP6ER1* also was associated with thiamethoxam resistance in BPH of China. *CYP6AY1* was found to be the most highly expressed gene of imidacloprid resistance BPH strain and functional studies provided evidence that the over-expression of *CYP6AY1* was contributing to imidacloprid resistance in the laboratory selected resistance strain^15^. However, using the same primers as previously reported, we found that *CYP6AY1* was down-regulated in many strains especially in thiamethoxam selected populations^5,15^. Screening of five field populations from China showed that *CYP6AY1* was only significantly overexpressed in one field population (SG2014, 13.5-fold, Fig. 5). Recent studies also showed that *CYP6AY1* was underexpressed in most of field collected populations compared with a lab susceptible strain^5^. It is possible that *CYP6AY1* is overexpressed in some *N*. *lugens* field populations in China and not the other places. Besides this, our study used a single reference lab susceptible strain to compared with obtain BPH field strains. Further experiments to investigate the relative roles of *CYP6ER1* and *CYP6AY1* in thiamethoxam and buprofezin resistance by comparing resistant strains with additional susceptible laboratory or field strains is required to confirm our findings.

### Transgenic expression of the potential resistance genes CYP6AY1 and CYP6ER1 in *Drosophila melanogaster*

**Figure 6:**
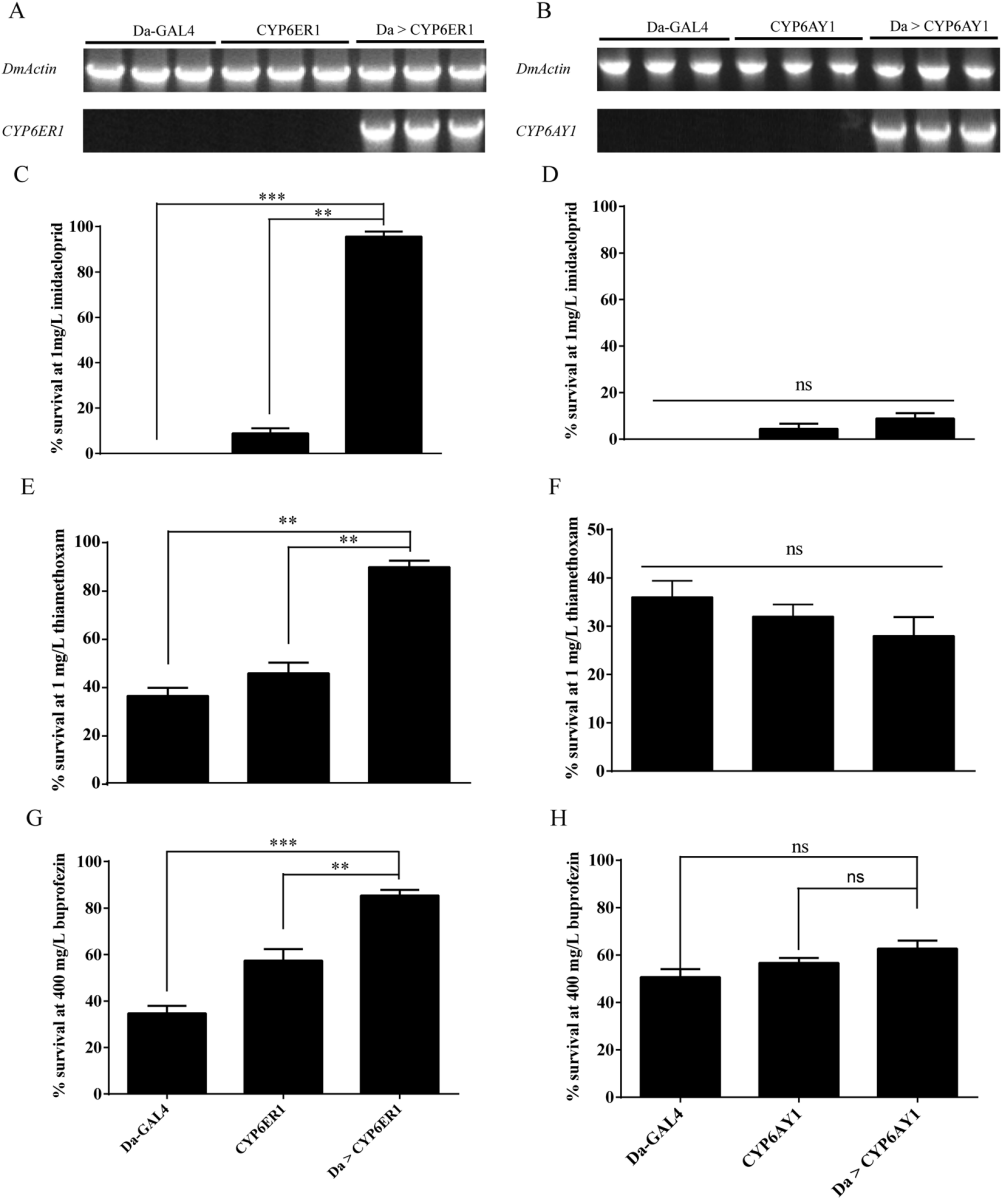
Transgenic expression of *CYP6ER1* (A,C,E and G) and *CYP6AY1* (B,D,F and H) in *D*. *melanogaster* and their effects on imidacloprid, thiamethoxam and buprofezin resistance. (**A** and **B**) The expressions of CYP6ER1 and CYP6AY1 were confirmed by RT-PCR in two control lines and transgenic line. Three biological replicates of Da-GAL4, flies with genetic background correspond to Da-GAL4; Three biological replicates of UAS-CYP6ER1 (A) or UAS-CYPAY1 (B), flies not expressing the CYP6ER1 or CYP6AY1; Three biological replicates of Da > CYP6ER1 (A) or Da > CYP6AY1 (B), transgenic flies expressing the CYP6ER1 or CYP6AY1. (**C,D,E,F,G** and **H**). The comparison between survival rates of two control lines and transgenic line exposed to 1 mg/ml imidacloprid (C and D), thiamethoxam (E and F) and 400 mg/ml buprofezin (G and H). The data shown are the mean ± s.e.m. (*n* = 3). **P < 0.01, ***P < 0.001 (Chi-squared Test), ns (no significant).

To identify whether the expression of *CYP6AY1* and *CYP6ER1* is sufficient to confer imidacloprid, thiamethoxam and buprofezin resistance, we used a transgenic approach utilizing the GAL4/UAS system of *D*. *melanogaster*. We confirmed the expression of the transgene in the Da > *CYP6ER1* and Da > *CYP6AY1* by RT-PCR (Fig. 6A and B). Bioassays showed that Da > *CYP6ER1* line was resistant to imidacloprid, thiamethoxam and buprofezin at a treated dose of 1 mg/L imidacloprid and thiamethoxam or 400 mg/L buprofezin, with significant higher survival rate than the control lines (Fig. 6C,E and G). However, Da > *CYP6AY1* line showed no significant resistance to imidacloprid, thiamethoxam and buprofezin (Fig. 6D,F and H). These data suggest that the expression of *CYP6ER1* is sufficient for causing imidacloprid, thiamethoxam and buprofezin resistance. However, the expression of *CYP6AY1* might play little roles in imidacloprid, thiamethoxam and buprofezin resistance.

### RNA interference of CYP6ER1

**Figure 7:**
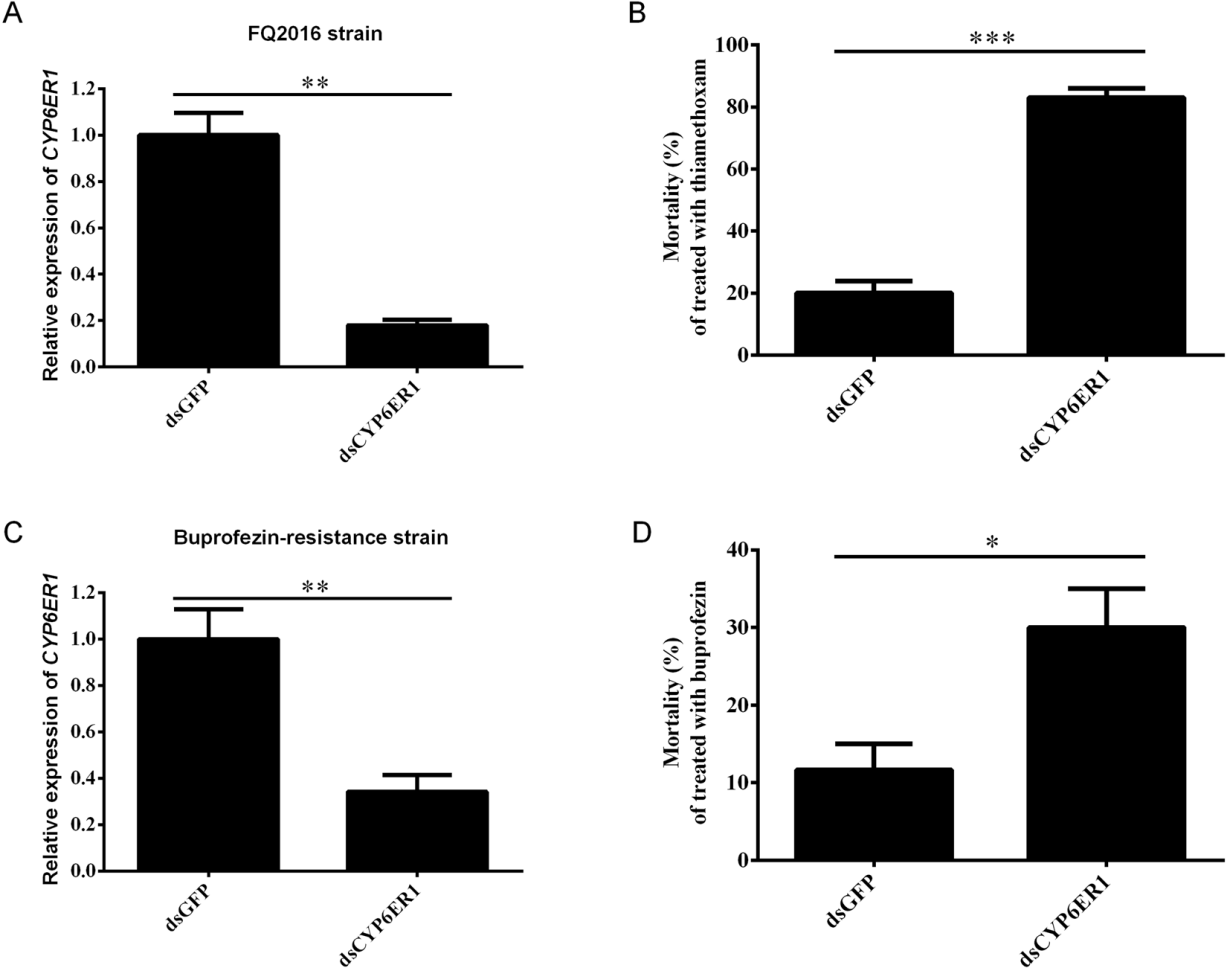
Knockdown in the expression of *CYP6ER1* in *N*. *lugens* field-resistance strain reduced its resistance to thiamethoxam and buprofezin. (**A** and **C**) The mRNA levels of *CYP6ER1* were quantified by qRT-PCR at three days after dsRNA injection in FQ2016 (A) or buprofezin-resistance (C) strains, respectively. The data shown are mean + SEM (*n* = 3). Statistical significance of the gene expression between two samples was calculated using Student’s *t* test. *P < 0.05, **P < 0.01 and ***P < 0.001. (**B** and **D**) Effects on the mortality rate after injection of *dsGFP* or *dsCYP6ER1* and followed by application of thiamethoxam (5 mg/L) (B) or buprofezin (50 mg/L) (D).

Recent work have showed that when *CYP6ER1* mRNA levels in imidacloprid-resistance strain was reduced by RNA interference (RNAi), imidacloprid susceptibility was recovered^20^. To further evaluate the contributions of *CYP6ER1* in thiamethoxam and buprofezin resistance *in vivo*, we designed dsRNA to silence *CYP6ER1* in 2^nd^ nymph from the FQ2016 and Bup-R strain, respectively. Our RNAi experiment showed that the *CYP6ER1* mRNA levels decreased by >80% or >60% in FQ2016 or buprofezin-resistance strains at 3 days after injection of *CYP6ER1* dsRNA, respectively, indicating that this gene was successfully silenced by RNAi (Fig. 7A and C). The mortality of the *dsCYP6ER1* sample was 60% and 20% higher than those of the *dsGFP* control sample after thiamethoxam and buprofezin treatment, respectively (Fig. 7B and D). These data suggests that the expression of *CYP6ER1* is required for the high level of thiamethoxam and buprofezin resistance in BPH.

## Conclusions

In summary, this study characterized resistance monitoring of brown planthopper populations of different geographic areas to eight insecticides using rice stem dipping method. The results provide current resistance status of insecticides against this economically important insect in China. There is evidence that individual planthoppers may exhibit multiple resistance to the different insecticide modes of action. Our results reveal that metabolism mediated by cytochrome P450 monooxygenases and overexpression of the cytochrome P450 *CYP6ER1* is associated with imidacloprid, thiamethoxam, and buprofezin resistance in *N*. *lugens* populations in China. Besides confirming this conclusion, we found that *CYP6ER1* was also involved in thiamethoxam and buprofezin resistance. Finally, although our studies provide evidence of a role for *CYP6ER1* in thiamethoxam and buprofezin resistance, functional characterisation of this P450 to confirm its ability to detoxify thiamethoxam and buprofezin is emergent required.

## Methods

### Insects

Seventy field populations of the brown planthopper were collected from seven provinces and the City of Shanghai in China from 2012 to 2016 (Fig. 1 and Supplementary Table S1). The collected insects were reared on rice seedlings under standard conditions of 27 ± 1 °C and 70–80% relative humidity with a 16-h light/8-h dark photoperiod. The field-collected brown planthoppers were mass mated, and the third-instar nymphs of the first (F1) or second (F2) generation were used for the susceptibility bioassay.

### Insecticides and synergists

Technical grade insecticides except sulfoxaflor were used in this study. Chlorpyrifos (96.5%), imidacloprid (97%) and nitenpyram (95%) were both supplied by Nanjing Red Sun Co. Ltd. (Jiangsu, China). Buprofezin (97%), thiamethoxam (98%), flufiprole (90%), pymetrozine (97.4%) and sulfoxaflor (22% SC) were supplied by Changlong Chemical Industrial Group Co. Ltd. (Jiangsu, China), Syngenta Investment Co. Ltd. (Shanghai, China), Dalian Ruize Chemicals Co. Ltd. (Liaoning, China), Aijin Agrochemical Co., Ltd. (Nanjing, China), and Dow AgroSciences China Ltd. (Shanghai, China), respectively; diethylmaleate (DEM) and triphenyl phosphate (TPP) by Shanghai Chemical Factory (Shanghai, China); and piperonyl butoxide (PBO) by Koch-Light Laboratories Co., Ltd. (UK). The technical grade insecticides were dissolved in acetone (*N*,*N*-dimethylformamide for pymetrozine) as stock solution and then diluted in a series of 5–6 concentration gradients with water containing 0.1% of Triton X-100 as described previously^3^.

### Bioassays

The dose-responses of BPH to different insecticides were measured using the rice-stem dipping method^19^. Rice plants at the tillering to early booting stage were pulled out from the soil, washed thoroughly, cut into an approximately 10-cm-long rice stem with roots and air-dried. Three rice stems were grouped together and dipped into appropriate insecticide solutions for 30 s and then air-dried at room temperature for at least 30 min. The rice stems with roots were wrapped with moistened cotton and put into 500 mL plastic cups. The third instar nymphs were collected with a homemade aspirating device, and twenty nymphs were transferred onto rice stems into a plastic cup for each replicate. There were three replicates for each concentration and 5–6 doses for each insecticide. Control rice stems were treated with 0.1% Triton X-100 water solution only. All treatments were maintained at a temperature of 27 ± 1 °C and 70–80% relative humidity with a 16-h light/8-h dark photoperiod. Mortality was recorded 72 h after treatment for chlorpyrifos, 96 h for imidacloprid, thiamethoxam, sulfoxaflor, flufiprole, nitenpyram, 120 h for buprofezin, and 168 h for pymetrozine according to the speed of kill of the insecticides. The nymphs were considered dead if they were unable to move after a gentle prodding with a fine brush.

For synergism bioassays of BPH to imidacloprid, thiamethoxam and buprofezin, third-instar nymphs were first exposed to rice stems treated with acetone solutions of the selected detoxification enzyme inhibitors at the highest possible concentrations (no adverse effect on the insects; PBO, P450-monoxygenase inhibitor at 20 mg/L; TPP, esterase inhibitor at 100 mg/liter; and DEM, glutathione S-transferase inhibitor at 50 mg/L determined by preliminary testing) for 2 h. The rest of the procedures were kept the same with above mentioned bioassay methods.

Three of the field strains, HX2015, DY2015 and XG2015, demonstrating relatively high levels of resistance to thiamethoxam, were placed directly onto rice plants treated with 40, 80 and 80 mg/L thiamethoxam and selected one generation in the laboratory.

### Data analysis

The mortality rates at diagnostic concentrations were subjected to Abbott’s formula. Lethal concentration values (LC_50_) and their 95% fiducial limits (F.L.) were estimated using POLO-Plus program (Version 2.0) (LeOra Software 2008). The resistance ratio (RR) was calculated by dividing the LC_50_ value of a field population by the corresponding LC_50_ value of the susceptible baseline (Supplementary Table S2). Synergistic ratio (SR) was calculated by dividing the LC_50_ value without a synergist treatment by the LC_50_ with a synergist treatment. Moreover, the LC_50_ value of the susceptible baseline for sulfoxaflor to *N*. *lugens* was established in the present study using a susceptible strain of *N*. *lugens*, which had been provided from the Zhejiang Chemical Industrial Group Co. Ltd. (Hangzhou, Zhejiang, China) and reared on rice-seedlings in the laboratory without exposure to any insecticide for many years. Insecticide resistance of the field strains was classified as: RR < 5-fold as susceptible, RR = 5~10-fold as low resistance level, RR = 10~100-fold as medium resistance level and RR > 100-fold as high resistance level^25^.

### Real-time quantitative RT PCR

Real-time quantitative RT PCR (qRT-PCR) was performed to determine the mRNA levels of P450s using the SsoFast Eva Green Supermix with Low Rox (Bio-Rad, Hercules, CA) and Applied Biosystems 7500 Real-Time PCR System (Applied Biosystems by Life Technologies, Carlsbad, CA) following the manufacturer’s instructions. Each qRT-PCR experiment was performed at least four independent biological replicates and analyzed in three technical replications. Data were analysed according to the 2^−ΔΔCT^ method^40^. The primers used in this study are designed based on previous report (Supplementary Table S3)^15,18^.

### Construction of transgenic *Drosophila* and bioassays

The *CYP6ER1* and *CYP6AY1* CDS cloned were inserted into a pJFRC-MUH vector to prepare UAS-*CYP6ER1* and UAS-*CYP6AY1* constructs, respectively. Subsequently, the F1 of “BDSC#8622 (y^1^w^67*c*23^; P(CaryP) attP2) male crossed to PhiC31 source virgin” were used for injection using standard techniques in the UniHuaii Co., Ltd. (Zhuhai, Guangdong, P.R. China). The transformed lines were backcrossed with W^1118^ line with several generations and then were crossed with the Da-GAL4 (Daughterless-GAL4, which expressed in all of the cells) line for expression of the *CYP6ER1* and *CYP6AY1* gene, respectively. The genotype of the crosses were Da-GAL4 > *CYP6ER1* and Da-GAL4 > *CYP6AY1*. For use as a control, the transformed line and the Da-GAL4 line were crossed with W1118 line, the progenies of which did not express *CYP6ER1* and *CYP6AY1* gene. RT-PCR was used to confirm the expression of the *CYP6ER1* and *CYP6AY1* gene in transgenic *Drosophila* using primers specific for the *CYP6ER1* and *CYP6AY1* gene and the reference housekeeping gene DmActin (Supplementary Table S3)^20^. Insecticide bioassays for imidacloprid and thiamethoxam were performed using previously described techniques^41^. In brief, 50 first instar larvae were placed in vials with 10 ml corn meal medium containing 1 mg/L imidacloprid and thiamethoxam or 400 mg/L buprofezin. Three to five replicates were performed for each assay. Adult emergence of 50 first instar larvae per vial was scored. The survival rates were calculated and analyzed using the Chi-squared test.

### RNA interference and bioassays

The coding sequences of *CYP6ER1* and green fluorescent protein (GFP) (negative control) were cloned into the pGEM^®^-T easy vector (Promega) vector. To minimize non-target silencing, a unique region of *CYP6ER1* gene was chosen for the design of dsRNAs. PCR-generated DNA templates were then used to synthesize dsRNA, which contains T7 promoter sequences at each end (Supplementary Table S3). We used a MEGAscript T7 transcription kit (Ambion, Austin, TX, USA) to produce the specific dsRNA of each gene as the manufacturer’s instruction. The quality and size of the dsRNA products were verified by 1% agarose gel electrophoresis. The 2^nd^ instar nymph were used for microinjection with 30 nymphs used for each gene treatment. Approximately 30 nl of purified dsRNA (5 ng/nl) was injected into the mesothorax of the nymph. A set of six to 10 insects at 3 days after injection for each treatment was selected to verify dsRNA knockdown efficiency by qRT-PCR. Thirty nymphs were placed in rice seedlings soaked in a solution of 5 mg/L thiamethoxam and 50 mg/L buprofezin for 30 seconds. The rest of the procedures were kept the same with above mentioned bioassay methods. Each experiment was repeated in triplicate.

## Electronic supplementary material

Dataset 1

Dataset 2
