# Supplementary material for: The evolution of insecticide resistance in the brown planthopper (Nilaparvata lugens Stål) of China in the period 2012–2016
Source: Sci Rep. 2018 Mar 15;8:4586. doi: 10.1038/s41598-018-22906-5 (PMC5854692; doi:10.1038/s41598-018-22906-5)
Supplement: Supplementary file 1 — Dataset 1 [file 41598_2018_22906_MOESM1_ESM.doc]

**The evolution of insecticide resistance in the brown planthopper (*Nilaparvata lugens* Stål) of China in the period 2012–2016**

**Supplementary Information**

**Author affiliation:**

**Shun-Fan Wu**

College of Plant Protection, State & Local Joint Engineering Research Center of Green Pesticide Invention and Application, Nanjing Agricultural University, Nanjing 210095, China.

**Bin Zeng**

College of Plant Protection, State & Local Joint Engineering Research Center of Green Pesticide Invention and Application, Nanjing Agricultural University, Nanjing 210095, China.

**Chen Zheng**College of Plant Protection, State & Local Joint Engineering Research Center of Green Pesticide Invention and Application, Nanjing Agricultural University, Nanjing 210095, China.

**Xi-Chao Mu**

College of Plant Protection, State & Local Joint Engineering Research Center of Green Pesticide Invention and Application, Nanjing Agricultural University, Nanjing 210095, China.

**Yong Zhang**

College of Plant Protection, State & Local Joint Engineering Research Center of Green Pesticide Invention and Application, Nanjing Agricultural University, Nanjing 210095, China.

**Jun Hu**

College of Plant Protection, State & Local Joint Engineering Research Center of Green Pesticide Invention and Application, Nanjing Agricultural University, Nanjing 210095, China.

**Shuai Zhang**

National Agro-tech Extension and Service Center, Ministry of Agriculture, Beijing 100125, China.

**Cong-Fen Gao**

College of Plant Protection, State & Local Joint Engineering Research Center of Green Pesticide Invention and Application, Nanjing Agricultural University, Nanjing 210095, China.

**Jin-Liang Shen**

College of Plant Protection, State & Local Joint Engineering Research Center of Green Pesticide Invention and Application, Nanjing Agricultural University, Nanjing 210095, China.

**Corresponding author:**

**Cong-Fen Gao**

College of Plant Protection, State & Local Joint Engineering Research Center of Green Pesticide Invention and Application, Nanjing Agricultural University, Nanjing 210095, China.

Email: [gaocongfen@njau.edu.cn](mailto:gaocongfen@njau.edu.cn).

## Supplementary data

**Table S1** Collection informations of *Nilaparvata lugens* from different prefectures

| Origins | | Populations | Date | Coordinates | Stage |
| --- | --- | --- | --- | --- | --- |
| Province | Prefecture |
| Zhejiang | Jinhua | JH12 | 15 Aug. 2012 | 28°90′N, 119°81′E | Nymphs |
|  |  | JH13 | 16 Aug. 2013 | 28°90′N, 119°81′E | Nymphs |
|  |  | JH14 | 21 Aug. 2014 | 28°53′N, 119°48′E | Adults |
|  |  | JH15 | 23 Oct. 2015 | 28°53′N, 119°48′E | Adults |
|  | Jiaxing | JX12 | 15 Sep. 2012 | 32°27′N, 120°27′E | Nymphs |
|  |  | JX13 | 26 Sep. 2013 | 32°27′N, 120°27′E | Adults |
|  |  | JX14 | 15 Sep. 2014 | 32°27′N, 120°27′E | Adults |
|  |  | JX15 | 24 Sep. 2015 | 32°27′N, 120°27′E | Adults |
|  |  | JX16 | 11 Oct. 2016 | 32°27′N, 120°27′E | Adults |
|  | Wenzhou | WZ12 | 26 Sep. 2012 | 27°03N′119°37′E | Adults |
| Jiangsu | Gaochun | GC13 | 12 Sep. 2013 | 31°32′N, 118°87′E | Nymphs |
|  |  | GC14 | 7 Aug. 2014 | 31°19′N, 118°52′E | Adults |
|  |  | GC15 | 14 Sep. 2015 | 31°19′N, 118°52′E | Nymphs |
|  | Yangzhou | YZ12 | 10 Sep. 2012 | 32°23′N, 119°26′E | Nymphs |
|  |  | YZ13 | 13 Sep. 2013 | 32°40′N, 119°52′E | Adults |
|  | Yancheng | YC12 | 11 Sep. 2012 | 33°22′N, 120°07′E | Nymphs |
|  |  | YC13 | 11 Sep. 2013 | 33°22′N, 120°07′E | Adults |
|  |  | YC14 | 20 Sep. 2014 | 33°22′N, 120°07′E | Adults |
|  |  | YC16 | 13 Oct. 2016 | 33°22′N, 120°07′E | Adults |
|  | Nantong | NT12 | 31 Aug. 2012 | 32°01′N, 120°30′E | Nymphs |
|  |  | NT13 | 29 Sep. 2013 | 32°01′N, 120°30′E | Adults |
|  |  | NT14 | 6 Oct. 2014 | 32°01′N, 120°30′E | Adults |
|  |  | NT15 | 21 Oct. 2015 | 32°01′N, 120°30′E | Adults |
|  | Jurong | JR12 | 27 Aug.2012 | 31°95′N, 117°17′E | Adults |
|  |  | JR13 | 14 Sep.2013 | 31°95′N, 117°17′E | Adults |
|  |  | JR15 | 8 Oct.2015 | 31°95′N, 117°17′E | Adults |
|  | Danyang | DY13 | 14 Sep. 2013 | 32°00′N, 119°19′E | Adults |
|  |  | DY14 | 27 Sep. 2014 | 32°00′N, 119°19′E | Nymphs |
|  |  | DY15 | 8. Oct. 2015 | 32°00′N, 119°19′E | Adults |
| Jiangxi | Taihe | TH12 | 16 Sep. 2012 | 26°48′N, 114°52′E | Nymphs |
|  |  | TH13 | 18 Jul. 2013 | 26°48′N, 114°52′E | Nymphs |
|  |  | TH14 | 25 Sep. 2014 | 26°48′N, 114°52′E | Nymphs |
|  |  | TH15 | 29 Aug. 2015 | 26°48′N, 114°52′E | Adults |
|  |  | TH16 | 30 Sep. 2016 | 26°48′N, 114°52′E | Adults |
|  | Shanggao | SG12 | 23 Aug. 2012 | 28°13′N, 114°55′E | Adults |
|  |  | SG13 | 18 Aug. 2013 | 28°13′N, 114°55′E | Nymphs |
|  |  | SG14 | 5 Sep. 2014 | 28°13′N, 114°55′E | Nymphs |
|  |  | SG15 | 30 Aug. 2015 | 28°13′N, 114°55′E | Adults |
| Anhui | Hexian | HX12 | 21 Aug. 2012 | 31°25′N, 118°13′E | Nymphs |
|  |  | HX13 | 12 Sep. 2013 | 31°25′N, 118°13′E | Nymphs |
|  |  | HX15 | 29 Aug. 2015 | 31°25′N, 118°13′E | Adults |
|  | Qianshan | QS13 | 13 Sep. 2013 | 30°62′N, 116°53′E | Nymphs |
|  |  | QS14 | 9 Oct. 2014 | 30°62′N, 116°53′E | Nymphs |
|  |  | QS15 | 22 Sep. 2015 | 30°62′N, 116°53′E | Nymphs |
|  | Lujiang | LJ13 | 7 Sep. 2013 | 31°15′N, 117°16′E | Nymphs |
|  |  | LJ14 | 10 Sep. 2014 | 31°15′N, 117°16′E | Adults |
|  |  | LJ15 | 30 Sep. 2015 | 31°15′N, 117°16′E | Adults |
| Fujian | Yongan | YA12 | 10 Oct . 2012 | 25°58′N, 117°22′E | Adults |
|  |  | YA13 | 28 Jul . 2013 | 25°58′N, 117°22′E | Adults |
|  |  | YA14 | 11 Sep. 2014 | 25°58′N, 117°22′E | Adults |
|  |  | YA15 | 19 Sep. 2015 | 25°58′N, 117°22′E | Nymphs |
|  |  | YA16 | 26 Jul. 2016 | 25°58′N, 117°22′E | Adults |
|  | Fuqing | FQ13 | 12 Aug. 2013 | 25°10′N, 119°01′E | Nymphs |
|  |  | FQ14 | 4 Sep. 2014 | 25°10′N, 119°01′E | Nymphs |
|  |  | FQ15 | 7 Aug. 2015 | 25°10′N, 119°01′E | Adults |
|  |  | FQ16 | 25 Sep. 2016 | 25°10′N, 119°01′E | Adults |
| Hubei | Xiaogan | XG12 | 25 Aug. 2012 | 30°33′N, 113°32′E | Nymphs |
|  |  | XG13 | 21 Aug. 2013 | 30°33′N, 113°32′E | Adults |
|  |  | XG14 | 11 Sep. 2014 | 30°33′N, 113°32′E | Nymphs |
|  |  | XG15 | 11 Sep. 2015 | 30°33′N, 113°32′E | Nymphs |
|  |  | XG16 | 28 Sep. 2016 | 30°33′N, 113°32′E | Adults |
| Shanghai | Jinshan | JS14 | 11 Sep. 2014 | 30°75′N, 121°33′E | Adults |
|  |  | JS15 | 25 Sep. 2015 | 30°75′N, 121°33′E | Nymphs |
|  |  | JS16 | 9 Oct. 2016 | 30°75′N, 121°33′E | Adults |
|  | Pudong | PD12 | 27 Sep.2012 | 30°08N,121°27E | Nymphs |
|  | Pudong | PD16 | 12 Oct.2016 | 30°08N,121°27E | Adults |
| Guangxi | Nanning | NN13 | 31 Oct. 2013 | 22°50′N, 108°20′E | Nymphs |
|  |  | NN15 | 27 Oct. 2015 | 22°50′N, 108°20′E | Adults |
|  | Guilin | GL12 | 7 Aug. 2012 | 24°18′N, 109°45′E | Nymphs |
|  | Yongfu | YF13 | 1 Jul. 2013 | 24°99′N, 109°98′E | Adults |

**Table S2** The LC_50_ values of the susceptibility baseline of *Nilaparvata lugens* to eight insecticides.

|  | Insecticides | LC_50_ (95%CI) mg/L | Reference |
| --- | --- | --- | --- |
| Nicotinc acetylcholine receptor (nAChR) competitive modulators | Imidacloprid | 0.08 (0.05-0.11) | (Wang et al., 2008a) |
|  | Thiamethoxam | 0.11 (0.09-0.12) | (Wang et al., 2008a) |
|  | Nitenpyram | 0.47 (0.25-0.61) | (Wang et al., 2008a) |
|  | Sulfoxaflor | 0.49 (0.25-0.75) | This study |
| Inhibitors of chitin biosynthesis | Buprofezin | 0.08 (0.06-0.09) | (Wang et al., 2008b) |
| Acetylcholinesterase (AChE) Inhibitors | Chlopyrifos | 0.41 (0.37-0.45) | (Wang et al., 2013) |
| Chordotonal organ Transient Receptor Potential Vanilloid (TRPV) channel modulators | Pymetrozine | 2.57 (1.69-3.61) | (Wang et al., 2013) |
| GABA-gated chloride channel blockers | Flufiprole | 0.08 (0.06-0.10) | This study |

**Table S3** The primers used in this study.

| Primers | Primer sequence (5’-3’) |
| --- | --- |
| **For cDNA cloning** |  |
| NlCYP6ER1-compF | GTTCAACGTGAAACTCACTGCAA |
| NlCYP6ER1-compR | CTTCTGTCTTGTCAATGCCTTCT |
| NlCYP6AY1-compF | GTTATGGCCTCTATACTGCAAGT |
| NlCYP6AY1compR | CTAGTAGCTCAGGTTTGCACTTA |
| **For qRT-PCR** |  |
| Nlactin-F | TGGACTTCGAGCAGGAAATGG |
| Nlactin-R | ACGTCGCACTTCAGATCGAG |
| NlCYP6ER1-RTF | ATTCCGGTCTATGCGCTTC |
| NlCYP6ER1-RTR | TGGATTGGCGCTCTCTTACT |
| NlCYP6AY1-RTF | GCTGTTTCACTTCTTGAGACTCCG |
| NlCYP6AY1-RTR | GCTTGAGCTGCTATAACACTCTCG |
| **For RNAi** |  |
| DsCYP6ER1-F | TAATACGACTCACTATAGGGTGTGCTCAGCCCCTCATTC |
| DsCYP6ER1-R | TAATACGACTCACTATAGGGGGAAAGTTTGCGACGCCATT |
| DsGFP-F | TAATACGACTCACTATAGGGAAGGGCGAGGAGCTGTTCACCG |
| DsGFP-R | TAATACGACTCACTATAGGGCAGCAGGACCATGTGATCGCGC |
| **For validation of transgenic *Drosophila* lines** |  |
| NlCYP6ER1-XhoI-F | TTCAGGCGGCCGCGGCTCGAGCAAAATGTGGGAAAACTCGTGG |
| NlCYP6ER1-XbaI-R | CCTTCACAAAGATCCTCTAGACTAAGTATCTCTTGCTATT |
| NlCYP6AY1-XhoI-F | TTCAGGCGGCCGCGGCTCGAGCAAAATGATCGAGATTGGCTTG |
| NlCYP6AY1-XbaI-R | CCTTCACAAAGATCCTCTAGATCAAAGCCCTTCAACATCA |
| Dmactin5C-F | CACACCAAATCTTACAAAATGTGTGA |
| Dmactin5C-R | AATCCGGCCTTGCACATG |
| NlCYP6ER1F | GTTCAACGTGAAACTCACTGCAA |
| NlCYP6ER1R | CTTCTGTCTTGTCAATGCCTTCT |
| NlCYP6AY1F | GTTATGGCCTCTATACTGCAAGT |
| NlCYP6AY1R | CTAGTAGCTCAGGTTTGCACTTA |

**References:**

Wang, P., Ning, Z.P., Zhang, S., Jiang, T.T., Tan, L.R., Dong, S., Gao, C.F., 2013. Resistance monitoring to conventional insecticides in brown planthopper, *Nilaparvata lugens* (hemiptera: delphacidae) in main rice growing regions in China. Chin. J. Rice Sci. 27, 191-197.

Wang, Y., Chen, J., Zhu, Y.C., Ma, C., Huang, Y., Shen, J., 2008a. Susceptibility to neonicotinoids and risk of resistance development in the brown planthopper, *Nilaparvata lugens* (Stål) (Homoptera: Delphacidae). Pest Manag. Sci. 64, 1278-1284.

Wang, Y., Gao, C., Xu, Z., Zhu, Y.C., Zhang, J., Li, W., Dai, D., Lin, Y., Zhou, W., Shen, J., 2008b. Buprofezin susceptibility survey, resistance selection and preliminary determination of the resistance mechanism in *Nilaparvata lugens* (Homoptera: Delphacidae). Pest Manag. Sci. 64, 1050-1056.
